# Supplementary material for: Comparative multiomics analysis of cell physiological state after culture in a basket bioreactor
Source: Sci Rep. 2022 Nov 23;12:20161. doi: 10.1038/s41598-022-24687-4 (PMC9686226; doi:10.1038/s41598-022-24687-4)

23666068.1:27816909:27817372:-@NW_023666068.1:27814992:27815118:-@NW_023666068.1:27812146:27812

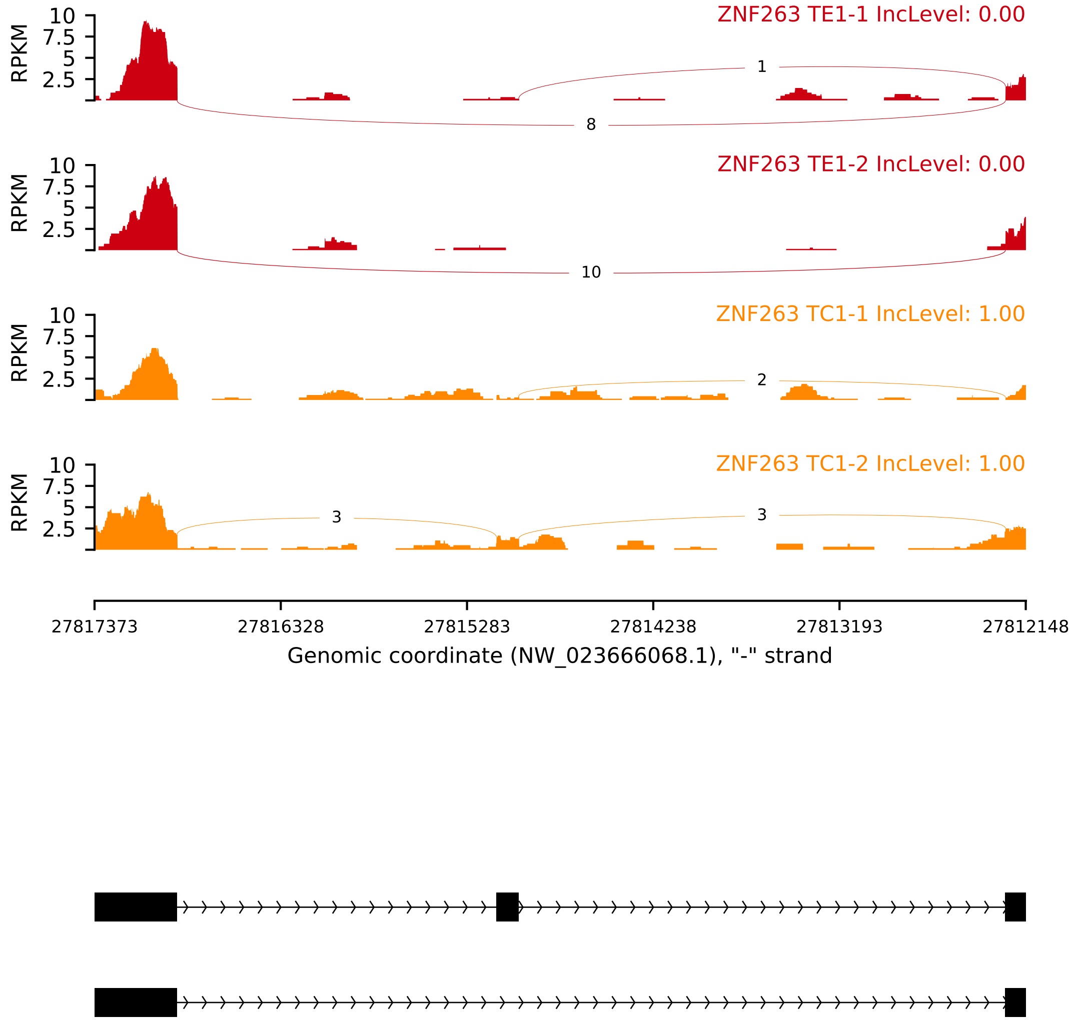

Supplement: Supplementary file 1 — Supplementary Information 1. [file 41598_2022_24687_MOESM1_ESM.zip › raw data/Transcriptomic raw data/7.AS/2.dasplot/TE1vsTC1/SE/18_ZNF263_NW_023666068.1_27816909_27817372_-@NW_023666068.1_27814992_27815118_-@NW_023666068.1_27812146_27812262_-.pdf]
